# Infective Endocarditis Presenting as Complete Heart Block With an Unexpected Finding of a Cardiac Abscess and Purulent Pericarditis

**DOI:** 10.14740/jocmr2228w

**Published:** 2015-09-25

**Authors:** Randolph E. Brown, John Michael Chua Chiaco, Jessica L. Dillon, Edward Catherwood, Kim Ornvold

**Affiliations:** a Department of Cardiology, Dartmouth-Hitchcock Medical Center, One Medical Center Drive, Lebanon, NH 03756, USA; b Department of Pathology, Dartmouth-Hitchcock Medical Center, One Medical Center Drive, Lebanon, NH 03756, USA

**Keywords:** Infective endocarditis, Myocardial abscess, Heart block, Purulent pericarditis

## Abstract

Intracardiac abscess resulting in complete heart block is an infrequent complication of infective endocarditis. Most presentations of endocarditis are limited to valvular and perivalvular structures, with varying degrees of heart block occurring in the minority of cases. We report a case of endocarditis manifesting as chest pain associated with ST segment elevation and complete heart block. The patient expired unexpectedly within a few hours of presentation. Postmortem examination revealed an atrial septal abscess, purulent pericardial collection, and fibrinous pericarditis. Spread of the abscess into the atrial septum was postulated to be the cause of the complete heart block. In endocarditis, the ominous development of heart block and a poor response to antibiotic therapy imply significant extension of the infection. Management therefore requires prompt ventricular pacing with consideration for valve replacement and possible pericardial drainage.

## Introduction

Myocardial abscess is a suppurative complication of infective endocarditis (IE) with potentially fatal outcomes. The precise incidence is unknown due to variations in reporting and best estimates can only be derived from IE figures. About 10,000 new cases of IE are diagnosed in the US each year, and of these, 30-40% are perivalvular abscesses. The aortic valve is the most commonly involved structure, followed by the mitral valve. The frequency of interatrial septal abscess is not known; however, five previous cases have been described after a review of the literature.

## Case Report

A 63-year-old Caucasian male with a history of substance abuse, metastatic liver cancer, hepatitis C, diabetes and peripheral arterial disease, presented to our emergency room with a 5-day history of atypical chest pain and dyspnea.

The severe chest pain was associated with light headedness and worsened with inspiration. There was mild dyspnea, but no change in his baseline abdominal swelling and pedal edema. He denied fever, cough and recent foreign travel.

Previously, this chronic smoker abused intravenous (IV) and intranasal illicit drugs. He was surgically treated for diabetic foot ulcers on several occasions and ultimately required a right below knee amputation. Two years prior to presentation, imaging for abdominal pain revealed metastatic hepatocellular cancer.

Upon presentation, the patient was jaundiced and cachectic with tense abdominal ascites and peripheral edema. He was hypotensive with a blood pressure of 81/45 mm Hg, bradycardic with a pulse of 41 bpm, afebrile, and maintaining adequate oxygen saturation on room air. His cardiopulmonary examination was unrevealing.

Labs were remarkable for leukocytosis, hyponatremia, mild acidosis and deranged liver function tests (Table 1).

**Table 1:** Pertinent Laboratory Data

Variable	Labs on admission	Reference range
Blood count		
Hemoglobin (g/dL)	12.8	13.7 - 17.5
White blood cell count (× 10^3^/μL)	17.3	4 - 10
INR	1.3	0.9 - 1.1
Chemistry		
Sodium (mmol/L)	123	135 - 145
Potassium (mmol/L)	5.1	3.5 - 5
HCO_3_ (mmol/L)	19	22 - 31
BUN (mg/dL)	32	10 - 20
Creatinine (mg/dL)	1.36	0.8 - 1.5
Glucose (mg/dL)	154	65 - 99
Lactate (mmol/L)	10.3	0.5 - 2.2
Liver function tests		
Total protein (g/dL)	2.3	0.2 - 1.3
Albumin (g/dL)	1.9	3.2 - 5.2
Total Bili (mg/dL)	2.3	0.2 - 1.3
Alk Phos (unit/L)	199	40 - 120
AST (unit/L)	67	0 - 39
ALT (unit/L)	71	0 - 55
Cardiac markers		
Troponin T (ng/mL)	0.1	< 0.03
Creatine kinase (unit/L)	89	0 - 200

INR: international normalized ratio; HCO_3_: bicarbonate; BUN: blood urea nitrogen; AST: aspartate aminotransferase; ALT: alanine aminotransferase.

**Figure 1:**
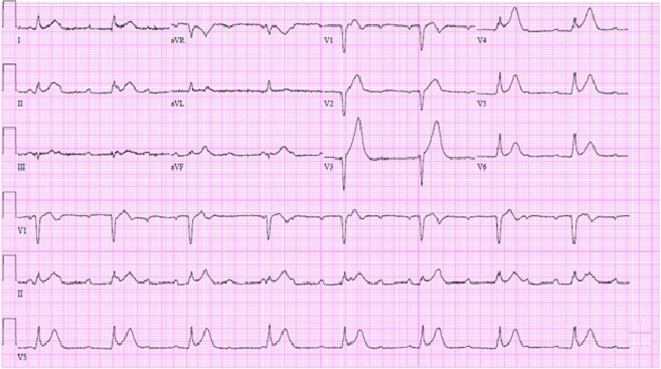
EKG in emergency room showing complete heart block with junctional escape at 48 bpm, Q waves in V1-V2 and diffuse ST segment elevations.

The patient’s electrocardiogram showed complete heart block with a junctional escape rhythm, an old septal infarct and diffuse ST elevations suggestive of pericarditis (Fig. 1). A limited echocardiogram revealed preserved left ventricular systolic function, no significant valvular disease and an unremarkable pericardium. Coronary angiography showed 75% left anterior descending stenosis and 50% right coronary artery stenosis. Because of the patient’s multiple comorbidities and suspicion that his symptoms were of a non-ischemic etiology, intervention was deferred.

The patient was transferred to the coronary care unit where a transvenous pacemaker was placed for the heart block. Blood cultures were drawn and a diagnostic paracentesis was performed for the possibility of sepsis.

The hypotension of 78/40 mm Hg was attributed to a combination of cirrhosis in the setting of possible sepsis. He was managed supportively with analgesics and IV fluid boluses. However, despite these efforts, the patient became unresponsive with pulseless electrical activity (PEA).

Cardiopulmonary resuscitation was immediately initiated with chest compressions and multiple doses of epinephrine. The ventricular fibrillation (VF) which soon followed, failed to respond to attempts at defibrillation. Ultimately, the patient eventually expired after 35 min of resuscitation.

Postmortem cultures of his ascitic fluid returned negative, but blood cultures were remarkable for methicillin-sensitive *Staphylococcus aureus* (MSSA).

**Figure 2:**
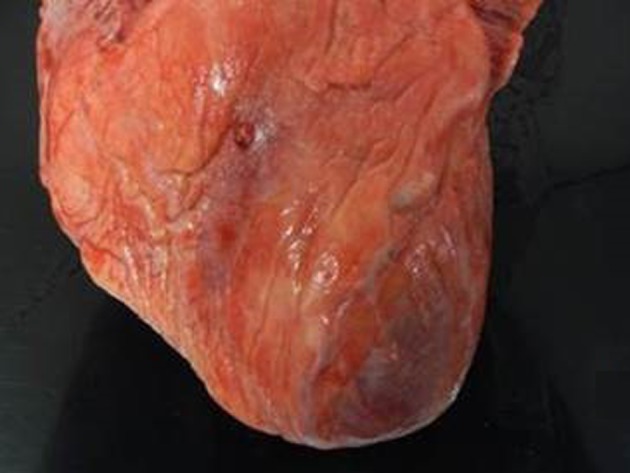
Gray exudate on granular epicardial surface consistent with fibrinopurulent pericarditis.

**Figure 3:**
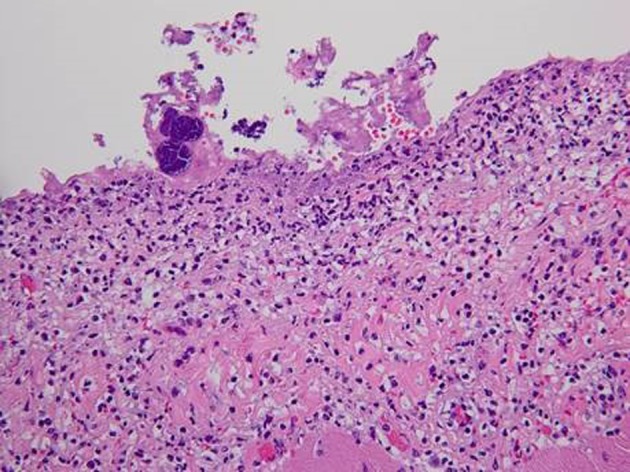
Photomicrograph (original magnification × 250) of the pericardium with severe inflammation, neutrophilic infiltration and fibrin with entrapped clusters of bacteria.

**Figure 4:**
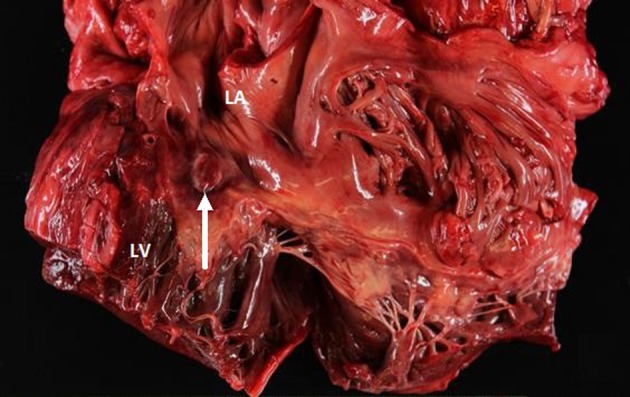
Gross view of the heart showing the abscess in the atrial septal wall (arrow). LV: left ventricle. LA: left atrium.

Postmortem examination revealed 85 mL of brown fluid in the pericardial sac. The visceral pericardium was roughened, granular, and microscopically demonstrated a fibrinopurulent exudate (Fig. 2). The pericardial sac contained neutrophils and clusters of coliform bacteria, consistent with purulent pericarditis (Fig. 3). The mitral valves were heavily calcified, but without vegetations. The atrial septum contained a 1.5 × 1.3 × 0.8 cm intramyocardial soft mass that spanned the full thickness of the septal wall (Fig. 4). On histologic examination, the myocardial mass displayed clusters of coliform bacteria, neutrophils, and dystrophic calcification, consistent with an intramyocardial abscess (Fig. 4). Additional pertinent autopsy findings include metastatic hepatocellular carcinoma to the adrenal glands bilaterally, portal vein thrombosis with tumor, and significant pulmonary congestion.

## Discussion

Historically, IE was seen in young adults as a complication of rheumatic heart disease or poor dental hygiene. Today, with an aging population, the higher prevalence of degenerative valvular disease, multiple comorbidities, drug abuse and the use of intracardiac devices, all contribute to the current epidemic of endocarditis [1].

The incidence of IE is about seven cases per 100,000 patient years [2], and these typically involve a perivalvular abscess of the aortic valve (41%) more so than the mitral valve (6%) [3]. In general, cardiac abscesses are seen in 30% of cases of native valve endocarditis and greater than 60% of cases of prosthetic valve endocarditis. However, the exact incidence of atrial septal abscesses is unknown. Our literature review has yielded five prior reports of perivalvular extension resulting in septal abscesses [4-6].

Complications of purulent pericarditis were seen in 22% of cases in one series [7] and complete heart block occurred in 11-14% of endocarditis patients [8]. Even though there have been several reports of endocarditis with a septal abscess and heart block, we found only two cases with an associated purulent pericarditis. A review of the literature has been summarized in Table 2 [4-6, 8-15].

**Table 2:** Infective Endocarditis With Heart Block: Review of the Literature

Case study	No. of cases	Age range	Comorbidities	Origin of infection	Bacteriology	Location of abscess	Valvular involvement	Heart block present	Outcome
Zettner and Irmiere, 1959 [4]	1	48	Schizophrenia	Suspected PNA	Culture negative	Atrial septum	MV/AV	CHB	Death
Holt et al, 1979 [5]	1	33	Bicuspid AV	Suspected PNA	*Staphylococcus aureus*	LA with fistula between AV and RA	AV	CHB (on digoxin)	Death
Langaker and Svanes, 1973 [6]	1	76	None	Gastroenteritis	*Salmonella typhimurium*	Between aorta and LA, atrial septum	MV	CHB (on digoxin)	Death
Kopelman et al, 1986 [8]	1	23	SLE, Recent Abortion	PID, Amniocentesis	Bacteroides	Aortic perivalvular, interventricular septum	MV/AV/TV	CHB	Death on POD #6 after AVR
Fordyce et al, 2011 [9]	1	72	Sciatica, A. Fib	Transrectal Prostate Biopsy	ESBL+ *E. coli*	None found on echo	TV	CHB	Successfully treated with antibiotics only
DiNubile et al, 1986 [10]*	211	12 - 88	N/A	N/A	Strep (50%), Staph (35%), Gram Neg Cocobacilli (5%), Gram Neg Bacilli (3%), fungi (1%)	None mentioned	AV (36%)/MV (33%)/TV (5%)	First Deg (45%), second Deg (15%), CHB (20%)	Death in 20% (43 cases), CHF in 7% of patients
Landi et al, 2009 [11]	1	64	Bicuspid AV, severe AS	Sepsis	*Staph aureus*	Aortic root abscess tracking into the RV free wall and the ventricular septum	AV/TV	None	Successfully treated with AVR and TVR
Wang et al, 1972 [12]*	142	49 - 77	Bicuspid AV, prosthetic valve	N/A	Strep (2%), Staph (1%), *E. coli* (1%)	WBC infiltration of atrial septum and AVN in one patient, cardioaortic fistula in five cases	AV (39%)/MV (27%)/AV + MV (20%)	CHB (4%) six patients, first Deg or second Deg (10%) 14 patients	Death in 40%, one successfully treated with AVR
Bacchion et al, 2007 [13]	1	46	Bicuspid AV, prosthetic valve	Dental abscess	Bacteroides fragilis	Perivalvular ring abscess of MV	AV/MV	First Deg and LBBB	Successfully treated with AVR and MVR
Chu et al, 2006 [14]	1	54	Alcoholic cirrhosis, variceal bleed, CKD	Infected arteriovenous shunt	Staph epidermidis	None found on echo	AV	CHB	Death
Park et al, 2011 [15]	1	38	Bicuspid AV, HTN	Recent dental procedure	*Staph aureus*	None found on echo	AV	First Deg	Successfully treated with AVR

*Noteworthy studies. AV: aortic valve; MV: mitral valve; TV: tricuspid valve; LA: left atrium; RA: right atrium; CHB: complete heart block; BBB: bundle branch block; AS: aortic stenosis; SLE: systemic lupus erythematosus; CKD: chronic kidney disease; PNA: pneumonia; AVN: atrioventricular node; AVR: aortic valve replacement; PID: pelvic inflammatory disease; POD: post-operative day; CHF: congestive heart failure; HTN: hypertension; A. Fib: atrial fibrillation; N/A: not applicable (missing data).

Review of prior case reports reveal that a number of comorbidities predispose to cardiac abscesses with heart block. Underlying conditions such as congenital heart disease, valve prosthesis, immunosuppression and endocardial defects due to turbulent blood flow all set the stage for endocarditis [16]. It is postulated that cardiac abscesses arise from the lodgment of infected emboli in coronary arteries [5, 6] or from contiguous spread from infected valves. The fact that the aortic and mitral valves share a common ring in part of their circumference and their proximity to the atrial septum explains the pattern of spread from a valvular abscess. According to Holt et al, myocardial abscesses are usually small and widespread throughout the myocardium. They are often underappreciated in IE and rarely occur in the septum.

The conduction system is contained within the right atrium and membranous septum. The left bundle branch runs along the base of the membranous septum or along the left side of the interventricular septum. Additionally, the mitral valve is located near the atrioventricular node. This may explain why abscesses of the posterior aortic sinus account for most cases of complete AV block in endocarditis [8]. Heart block is associated with 36% of aortic and 33% of mitral valve diseases [9]. There are no meta-analyses of IE with heart block. However, in a review of 142 cases of endocarditis by Wang et al, they discovered six cases (4%) of complete heart block and 14 cases (10%) of first or second degree heart block were reported. DiNubile et al, on the other hand, studied 211 cases of endocarditis and found that 20% had a complete heart block [10].

In the pre-antibiotic era, purulent pericarditis was due primarily to various infections such as pneumonia and endocarditis. Currently, the most common predisposing factors for hematogenous and direct contiguous spread include immunosuppression, cardiac surgery, trauma, renal disease on dialysis and substance abuse [17]. We were able to uncover two additional cases of purulent pericarditis and endocarditis. The first occurred in a 76-year-old patient with salmonella gastroenteritis who perished [6] and the second in an 18-month-old boy [18] who was successfully treated.

### Conclusion

Our case is relatively unique in that our patient presented with an atrial septal abscess, complete heart block and a purulent pericarditis. The culture-positive *Staphylococcus aureus* in our patient is probably related to his history of IV drug use or secondary seeding from his infected amputated right leg, as streptococcus is usually more commonly found in native valve endocarditis.

This case was unusual in that, compared to other reports, there were no physical exam findings suggestive of endocarditis. The mild leukocytosis and absence of fever could be explained by his uncontrolled diabetes and metastatic cancer. However, the diffuse concave upward ST elevations on presentation were consistent with pericarditis.

In general, myocardial abscess should be suspected if there is poor response to antibiotic therapy. The development of heart block during the course of endocarditis indicates extension of the infection and implies a poor prognosis [5]. Management should therefore consist of appropriate antibiotics, ventricular pacing, surgical valve replacement and consideration for prompt pericardial drainage.
